# Identifying disease-gene associations using a convolutional neural network-based model by embedding a biological knowledge graph with entity descriptions

**DOI:** 10.1371/journal.pone.0258626

**Published:** 2021-10-15

**Authors:** Wonjun Choi, Hyunju Lee

**Affiliations:** School of Electrical Engineering and Computer Science, Gwangju Institute of Science and Technology, Buk-gu, Gwangju, Republic of Korea; Taipei Medical University, TAIWAN

## Abstract

Understanding the role of genes in human disease is of high importance. However, identifying genes associated with human diseases requires laborious experiments that involve considerable effort and time. Therefore, a computational approach to predict candidate genes related to complex diseases including cancer has been extensively studied. In this study, we propose a convolutional neural network-based knowledge graph-embedding model (KGED), which is based on a biological knowledge graph with entity descriptions to infer relationships between biological entities. As an application demonstration, we generated gene-interaction networks for each cancer type using gene-gene relationships inferred by KGED. We then analyzed the constructed gene networks using network centrality measures, including betweenness, closeness, degree, and eigenvector centrality metrics, to rank the central genes of the network and identify highly correlated cancer genes. Furthermore, we evaluated our proposed approach for prostate, breast, and lung cancers by comparing the performance with that of existing approaches. The KGED model showed improved performance in predicting cancer-related genes using the inferred gene-gene interactions. Thus, we conclude that gene-gene interactions inferred by KGED can be helpful for future research, such as that aimed at future research on pathogenic mechanisms of human diseases, and contribute to the field of disease treatment discovery.

## Introduction

Cancer is a major threat to public health, with over 18.1 million new cases and 9.6 million cancer deaths in 2018. Lung cancer is the most commonly diagnosed cancer (11.6% of total cases) and is the leading cause of cancer death (18.4% of total cancer deaths). This is closely followed by female breast cancer (11.6%) and prostate cancer (7.1%) [1]. Cancer is a genetic disease, and cancer-related genes are mutated and dysregulated, leading to tumor formation and cancer [2]. As genes function together in signaling and regulatory pathways, somatic mutations and changes in RNA and protein expression result in abnormal gene-gene interactions. It is therefore essential to understand cancer-related genes in the context of a gene-gene interaction network to enhance our knowledge about cancer development.

Over recent decades, the advancement of next generation sequencing technologies [3] and microarray development [4] have encouraged many studies for identifying the major genes involved in the physiopathology of various diseases, including cancer. These technologies and studies allow us to monitor gene activity in cells [5, 6], leading to many scientific articles demonstrating associations between diseases and genes. In addition, exome and genomic sequencing studies have demonstrated that different patterns of somatic mutation and gene expression changes affect cancer initiation and progression [7]. Thus, recent computational algorithms [8, 9] have exploited mutations in genes to discover new cancer-associated genes from genome sequencing data. In another previous work [10], researchers utilized both mutation information of a given gene as well as that of its neighbors in a functional network. These efforts made possible to discover new cancer-related genes, and newly discovered information can thus be accumulated in the literature.

The last few decades have witnessed massive advances in biomedical research, resulting in valuable knowledge and large amounts of data that are continuously updated and stored in various freely accessible databases for further research. For example, the Comparative Toxicogenomics Database (CTD) [11] integrates data from the scientific literature that can be searched by professional biocurators to describe chemical interactions among genes, in addition to identifying associations between diseases and genes or chemicals. BioGRID [12] is a well-known protein-gene interaction database that is manually curated from literature in the Medline database. As the identification of genes associated with human diseases requires laborious experiments that involve considerable effort and time, computational approaches that utilize existing biological knowledge contained in such databases will be helpful for automatically predicting the undiscovered genetic pathogenesis of cancer. In addition, gene-gene interaction networks have been constructed for humans, and have been used as an informative resource for computationally predicting cancer-related genes [13, 14].

**Fig 1:**
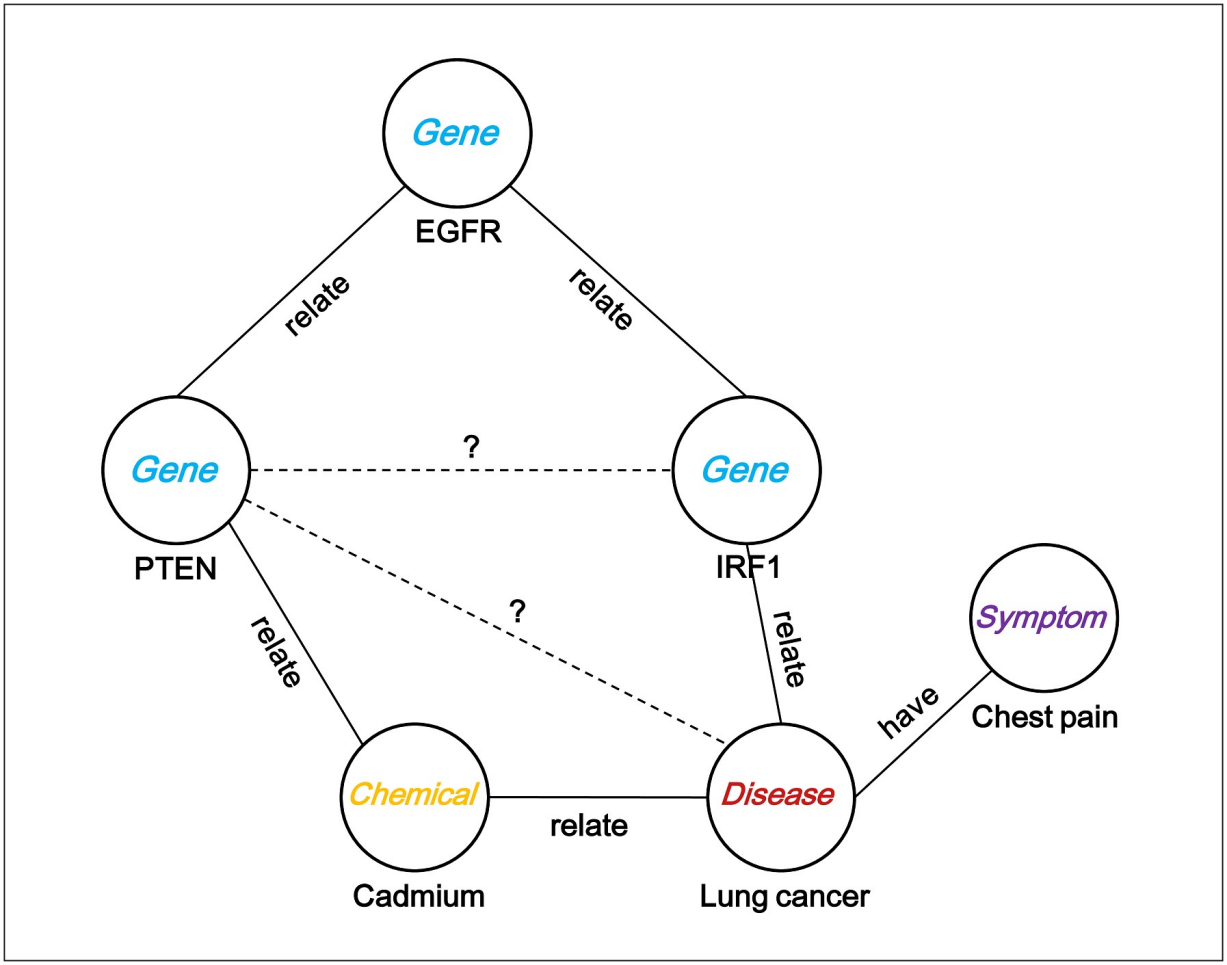
A toy example of inferring new relationships from existing knowledge bases. The knowledge graph in the toy example contains relationships between biological entities. The relationship between PTEN and Lung cancer can be inferred from the information on the relationship between PTEN and cadmium and between cadmium and lung cancer. Also, the relationship between PTEN and IRF1 can be predicted by the relationship between PTEN and EGFR and between EGFR and IRF1.

Knowledge graphs organize human knowledge into structured information in the real world, and are important resources for intelligent applications such as question answering, personalized recommendation systems, machine translation, and web searches [15]. A typical knowledge graph consists of a collection of knowledge bases (KBs or fact triples) representing relationships between two entities. KBs or triples are in the form of (*head entity*, *relation*, *tail entity*). Since a knowledge graph is a way of connecting the fragmented knowledge, undiscovered knowledge can be inferred from the graph. For example, relationships denoted as dashed lines in Fig 1 can be predicted by existing knowledge bases in the graph. Over the past several decades, the content and volume of general-purpose KBs, such as FreeBase [16], WordNet [17], YAGO [18], DBpedia [19], and Wikidata [20] has rapidly accumulated due to collaborative contributions by experts and the public. With the growing amount of KBs, many knowledge graph embedding models, such as TransE [21] and ConvKB [22], have been proposed to infer new triples by embedding existing multi-relational data in a low-dimensional vector space. However, they have not been widely applied and evaluated in biological KBs for representing relationships between biological entities such as chemicals, genes, diseases, and symptoms because most knowledge graph embedding methods were originally designed for general-purpose KBs. In our previous study [23], we demonstrated that TransE is useful for inferring new biological relationships. However, the performance of TransE with biological data was not as satisfactory as that of general-purpose KBs.

**Fig 2:**
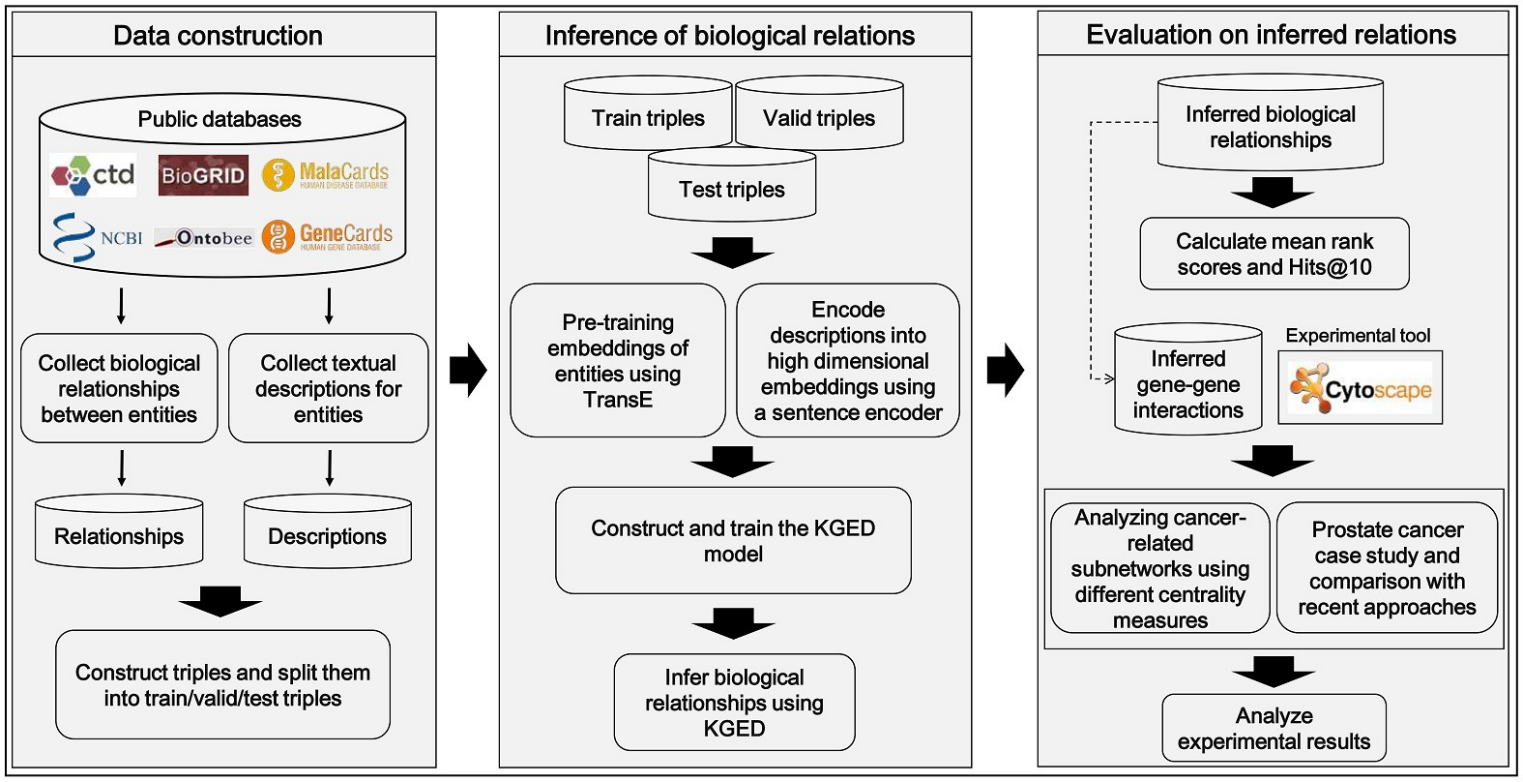
Summary of the complete study process. Biological knowledge bases (KBs) and their entity descriptions were collected from public databases. We then converted biological KBs into training, validation, and test triples using a dictionary for entities. Using these data, we learned the KGED model to infer biological relationships. Next, we calculated the mean rank scores and hits@10 scores to evaluate our model. We also performed additional experiments to prove the usefulness of gene-gene interactions inferred by KGED.

In this study, we propose a convolutional neural network (CNN)-based knowledge graph embedding model using the biological knowledge graph with entity descriptions (KGED) to identify disease-gene associations. Moreover, we compared the performance of KGED to that of TransE and ConvKB to demonstrate the superior performance of our system when embedding biological KBs. We then predicted a gene-interaction network related to each cancer type using KGED and analyzed the constructed gene network. We used network centrality measures to rank the central genes of the network and identify possible cancer-related genes. We evaluated our proposed approach for prostate, breast, and lung cancers by comparing it with existing approaches [13, 14, 24, 25]. A diagram explaining the entire procedure of this study is shown in Fig 2. The source codes for KGED and datasets used for biological KBs and entity descriptions are available at https://github.com/anispike1988/KGED.

## Materials and methods

### Biological knowledge bases and entity descriptions

**Biological knowledge bases and entity descriptions**. Knowledge graphs are graph-structured KBs, wherein facts are represented in the form of edges between nodes. As an entity may have multiple aspects and various relations may focus on different aspects of entities, these generally consist of different types of entities and relations. To maximize connectivity between entities, we constructed the biological knowledge graph using chemical-gene, chemical-disease, gene-gene, disease-gene, and disease-symptom relationships. These were obtained from public databases. A chemical-gene relationship indicates that a chemical upregulates or downregulates a gene, a chemical-disease relationship represents a chemical that is used to treat or cause a disease, and a disease-gene relationship indicates a gene targeted for the treatment of a disease or that the gene causes a disease. These three relationships were extracted from the CTD database (http://ctdbase.org/downloads/) as they provide files in tsv format for each relationship. Moreover, we downloaded a tab-separated text file containing the gene-gene interactions from the BioGrid database (https://downloads.thebiogrid.org/BioGRID). Lastly, we extracted disease-symptom relationships from the MalaCards human disease database (https://malacards.org/), an integrated compendium of annotated diseases mined from 72 meta-resources [26], by parsing xml files for each disease, which indicate that a disease contains diverse symptoms. We then integrated all files extracted from each database to obtain the biological triples. During the construction of the biological triples, only manually curated data by experts were used, due to their higher confidence levels than inferred data. Table 1 shows more details regarding the statistics of biological triples in terms of each relation. In this study, we used 3,273,215 triples with 103,625 entities, including 15,267 chemicals, 68,364 genes, 11,266 diseases, and 8728 symptoms, for training and evaluating the knowledge graph embedding models, as shown in Table 2.

**Table 1:** Statistics of biological triples between chemicals, genes, diseases, and symptoms obtained from the public databases.

Relations (*h, r, t*)	#Head entities	#Tail entities	#Triples	Data sources
Chemical, relate, gene	12,439	35,115	834,214	CTD
Chemical, relate, disease	9348	2973	89,457	CTD
Disease, relate, gene	5111	6760	27,363	CTD
Gene, relate, gene	49,590	49,590	2,193,026	BioGrid
Disease, have, symptom	9060	8728	129,155	MalaCards

The table means the details of biological dataset. The first column represents the data type. The second, third, and fourth columns represent the number of head and tail entities in each data type and triples, respectively. Data sources for each data type are described in the last column.

**Table 2:** Statistics of datasets used for training and evaluating the knowledge graph embedding models.

Data set	Biological KBs
#Entities	103,625
#Relations	5
#Triples	3,273,215
#Train	3,269,465
#Valid	2500
#Test	1250

We split our biological dataset into training, validation, test triples. 250 test triples and 500 validation triples for each data type were randomly selected and the rest of triples were used as training triples. They were used to evaluate the performance of knowledge graph embedding models.

**Fig 3:**
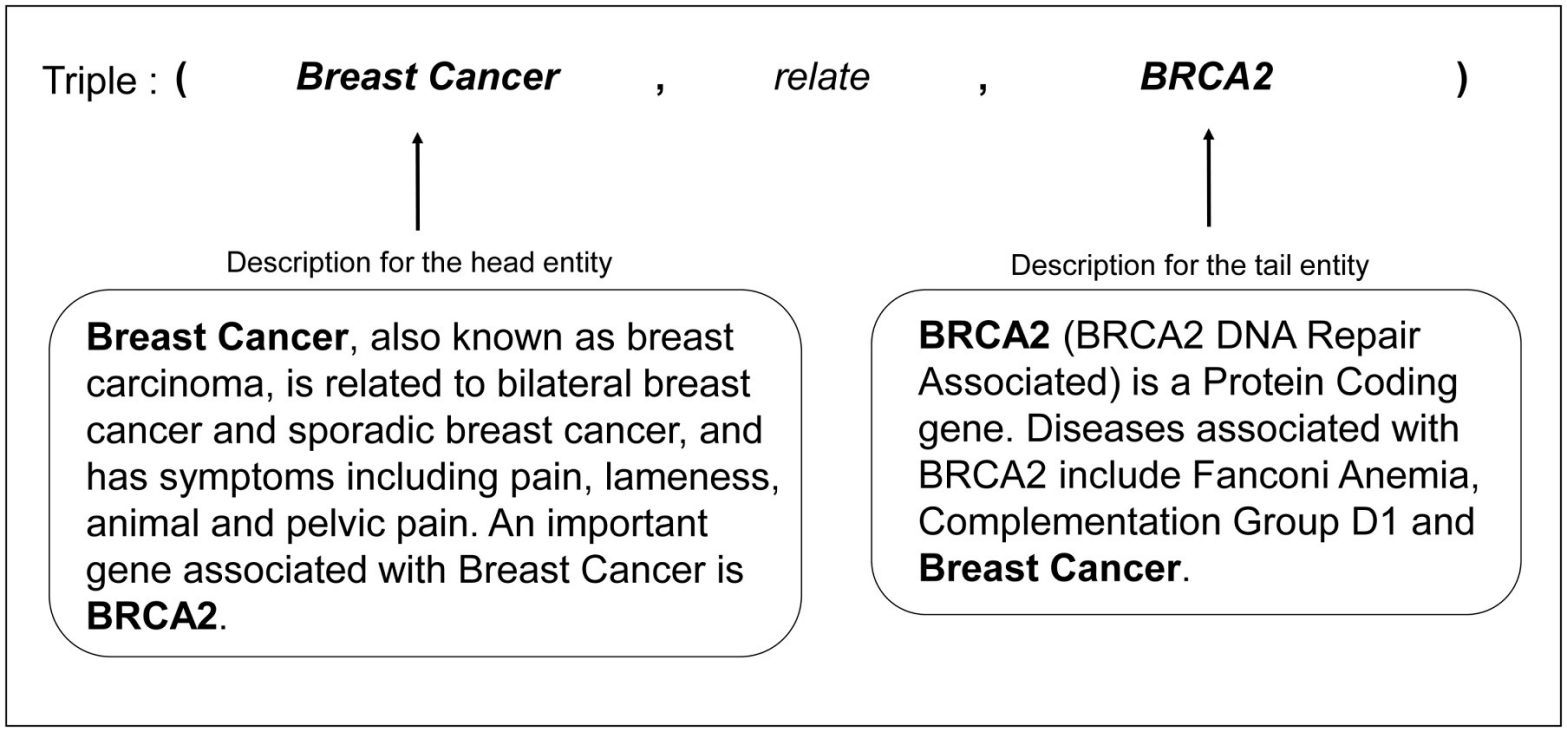
Example of entity descriptions for the disease (Breast cancer) and the gene (BRCA2). The textual description for breast cancer contains a strong semantic relationship between breast cancer and other diseases and genes. Also, the description for BRCA2 represents that the BRCA2 gene is related to breast cancer. This rich semantic information contributes to the biological knowledge graph.

Textual information can contribute to representation of the knowledge graph. In the biomedical domain, there are concise descriptions for biological entity terms. These consist of sentences, phrases, or short paragraphs with rich semantic information about these entities. Learning how to jointly embed with both fact triples and entity descriptions can greatly promote better knowledge acquisition and inference. For example, Fig 3 shows the descriptions of two entities in a fact triple. The description for the head entity, breast cancer, demonstrates a strong relationship between breast cancer and other diseases and symptoms. It also shows an association between breast cancer and the BRCA2 gene, which is the tail entity of the example triple. Furthermore, the description of the tail entity, BRCA2, represents a connection between the BRCA2 gene and diseases such as breast cancer. This rich semantic information contributes to the knowledge graph. Textual descriptions for diseases were obtained from the MalaCards database. We obtained xml files for each disease from the MalaCards database from which all descriptions for each disease were extracted. In addition, we extracted descriptions for symptoms and genes by parsing xml files collected from Ontobee (http://ontobee.org/), a web system serving as a linked data server and browser specifically targeted for ontology terms [27], and GeneCards (https://genecards.org/) [28], respectively. Lastly, we collected descriptions for chemicals using the E-Utilities API provided by the National Center for Biotechnology Information database [29]. To automatically obtain the descriptions for chemicals, we queried Medical Subject Heading (MeSH) identifiers of the chemicals to the API and then extracted only the description for the corresponding chemical.

**Fig 4:**
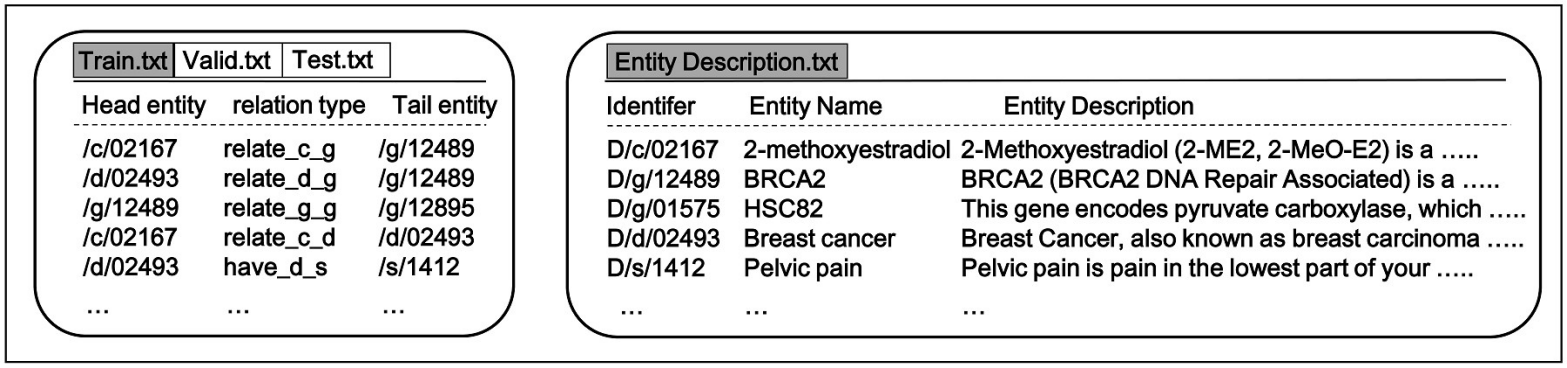
Example of data structures of biological KBs and their textual descriptions. Both biological KBs and their textual descriptions are stored in text formatted files. In the left box, biological KBs are split into training, validation, and test text files. The contents in these files consist of unique identifiers (IDs) of head and tail entities and relation types. In the right box, textual descriptions are stored. This file contains IDs for entity descriptions, entity names, and their descriptions. These files are used as inputs of the KGED model.

Fig 4 shows the data structure of the biological KBs and entity descriptions. The biological KBs in the training, validation, and test datasets are stored in a text formatted file. Each column represents unique IDs of the head entities, relation types, and IDs of tail entities, respectively. Textual descriptions for biological entities are also stored in the text file. Each column shows unique IDs, entity names, and their descriptions, respectively. Note that both datasets were used as inputs of our KGED model.

### Knowledge graph embedding models

A knowledge graph consists of a set of fact triples in the form of (*h*, *r*, *t*), where *h*, *t* are head and tail entities, and *r* indicates a relation between them (*e.g*., (*Elon Musk, Founded, Tesla*)). The vectors for (*h*, *r*, and *t*) are denoted as ***h***, ***r***, and ***t*** in bold italic, respectively. A knowledge graph embedding model aims to embed the knowledge graph into a continuous low-dimensional space, and continuous numerical vectors can be used to reflect the structural characteristics of the knowledge graph. There are various methods for embedding the general-purpose knowledge graphs such as FreeBase and WordNet. In this section, we introduce two knowledge graph embedding models.

TransE [21] is the first translation-based method which considers the relation as a translation vector ***r*** between two entity vectors ***h*** and ***t*** in a low-dimensional vector space. That is, the embedding vector (***h***+***r***) is close to ***t*** when the triple (*h*, *r*, *t*) holds. Therefore, the energy score function of TransE, *E*_r_(*h*, *t*), is expressed as follows:

$$\begin{array}{c} E_{r}(h,t)={\left\|h+r-t\right\|}_{L1/L2}. \end{array}$$ (1)

The energy score function is small if (*h*, *r*, *t*) exists, and is otherwise large. TransE is simple and efficient, and has performed well for 1:1 relations given its simplicity. However, the primary disadvantage of TransE is that it has problems with modeling complex relations such as 1:*N*, *N*:1, and *N*:*N*, where *N* is the number of entities.

ConvKB [22] proposes a novel use of a CNN to capture semantic information between entities and relations for knowledge graph embedding. In ConvKB, each entity or relation is transformed to a unique *k*-dimensional embedding. For each triple (*h*, *r*, *t*), *k*-dimensional embeddings (***h***, ***r***, ***t***) are represented as a *k* × 3 input matrix by concatenating each embedding vector. Thereafter, this matrix is fed to a convolution layer where different filters are used to extract multiple feature maps. These feature maps are then concatenated into a single feature vector, which is computed with a weight vector via a dot product to generate a score of the triple (*h*, *r*, *t*). This score is applied to estimate whether the triple is valid or not. The score function of ConvKB, *f*_r_(*h*, *t*), is defined as follows:

$$\begin{array}{c} f_{r}(h,t)=concat(g([h,r,t]*\Omega ))\cdot w \end{array}$$ (2)

where * denotes a convolution operator, **Ω** is the set of filters, *γ* is the number of filters, and $w\in R^{\gamma k\times 1}$ is a weight vector. In this paper, we used ConvKB as a baseline model to jointly embed representations of triples and biological descriptions for entities in the triples, and compared the performance of KGED with that of TransE and ConvKB.

### Entity normalization

Knowledge graph embedding models generally require a set of triples as an input. We therefore modified the biological dataset described in Table 1 into the form of a triple (*h, r, t*) to ensure that it functions properly in KGED. We assigned unique identifiers to each entity in our dataset. For example, the following disease-gene data, (*cardiomyopathies is related to CYCS*), can be denoted as the following disease-gene triple: (*/d/13364*, *d_relate_g*, */g/28644*). The following chemical-gene data, (*bisphenol a is related to MMS22L*), can be represented as the following chemical-gene triple: (*/c/11853, c_relate_g, /g/06856*). Before assigning unique identifiers to entities, we first normalized entity names for genes and diseases, as the use of these entity names could vary according to CTD, BioGrid, and MalaCards. This normalization task is necessary since such name variations can result in assigning different vector embeddings for some entities that are actually the same entities. To normalize disease names in our dataset, we first constructed a disease dictionary containing disease information, including disease names, concept identifiers such as MeSH [30], Online Mendelian Inheritance in Man (OMIM) [31], and ICD [32], in addition to synonyms using public databases. Based on the disease dictionary, we assigned unique identifiers to disease names in our dataset. We also built a gene dictionary containing gene symbols with corresponding identifiers and synonyms using CTD and BioGrid. Using this gene dictionary, we gave gene names in our dataset unique identifiers.

### Initialization of embeddings of triples and textual descriptions

As previously mentioned, KGED uses two types of inputs; one is the matrix for the triple, and the other is the matrix for the descriptions in terms of the corresponding triple. In [33], it has been shown that pre-trained embedding vectors can be used to achieve better generalization than random initialization. We therefore initialized the embeddings for entities ∈ ***E*** and a relation ∈ ***R*** for (*h*, *r*, *t*) in *T* by training TransE for 3,000 epochs. Textual descriptions for biological entities consist of sentences, phrases, or short paragraphs, and contain rich semantic information about these entities. We initialized the embeddings for entity descriptions *h*_*d*_, *t*_*d*_ ∈ ***ED*** by using universal sentence encoder [34], which is a model designed for encoding sentences into high dimensional vectors, surpassing the performance of word-level embeddings (e.g., Word2vec [35], Glove [36], and Fasttext [37]). The input of the encoder is variable length English text, and the output is a 512-dimensional vector. In this process, we manually generated descriptions for five relations as follows:

1. (chemical, relate, gene): “a chemical is related to a gene”
2. (chemical, relate, disease): “a chemical is related to a disease”
3. (disease, relate, gene): “a disease is related to a gene”
4. (gene, relate, gene): “a gene is related to a gene”
5. (disease, have, symptom): “a disease contains a symptom”.

Thereafter, the embeddings for relation descriptions *r*_*d*_ ∈ ***ER*** were also initialized by the universal sentence encoder. Consequently, we could obtain the pre-trained embedding matrices $A=[h,r,t]\in R^{k\times 3}$ and $B=[h_{d},r_{d},t_{d}]\in R^{l\times 3}$ as the inputs of KGED.

### Overall architecture of the proposed model

**Fig 5:**
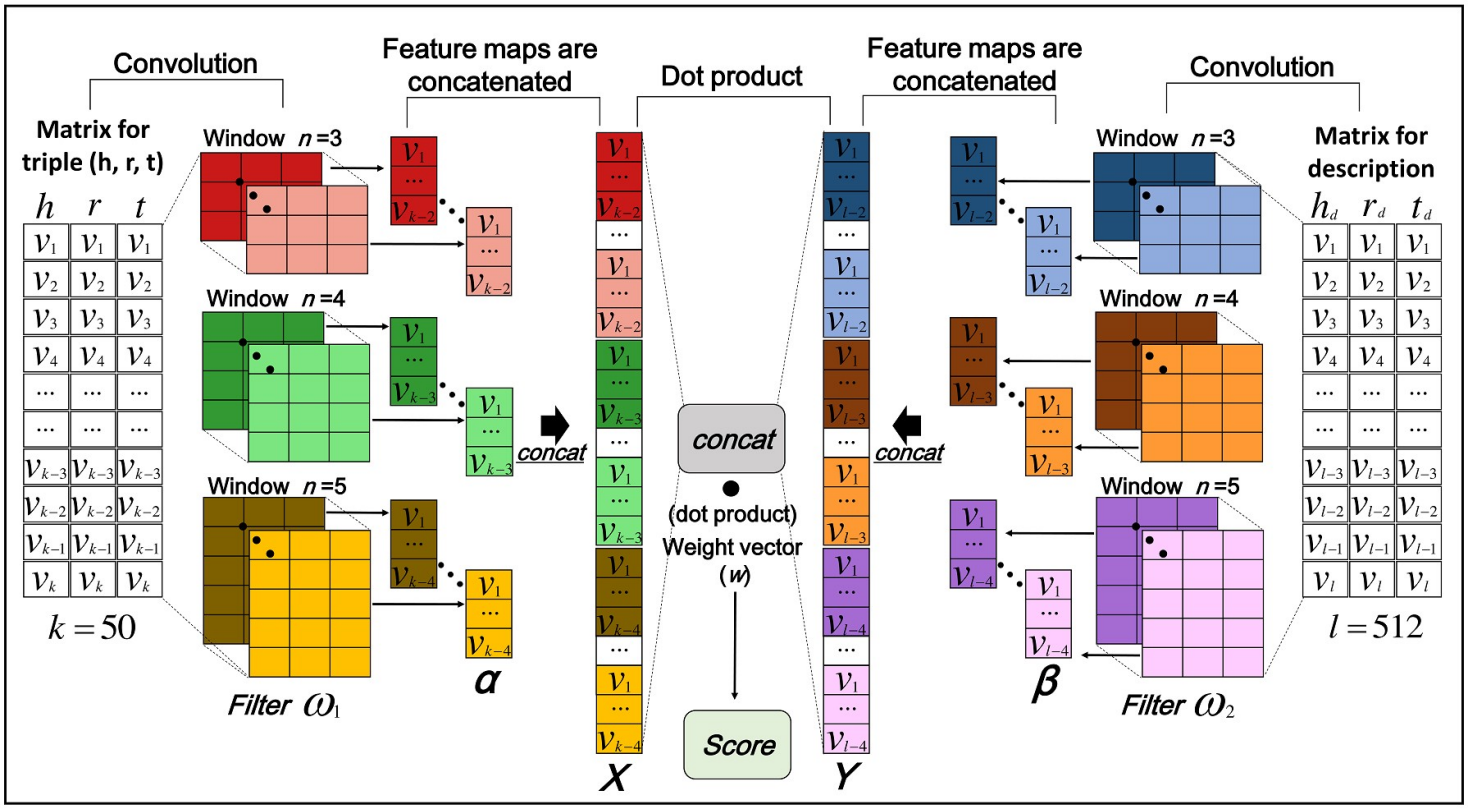
Overall architecture of KGED. In the KGED model, the input layer contains two parts; the first is the matrix for the triple, and the second is the matrix for the corresponding descriptions of each element in the triple. The former is initialized by pre-training TransE for 3,000 epochs. The latter is encoded by the universal sentence encoder to reduce the dimensionality. The filters (*ω*_1_, *ω*_2_) are then convolved with these two inputs to generate feature maps (*α*, *β*). Thereafter, all feature maps are concatenated to one vector, which can be the representation of the inputs. This vector is computed with a weight vector ***w*** via a dot product to give a score for the triple.

In this section, we introduce the model architecture of KGED, summarized in Fig 5. A KB is a collection of fact triples in the form of (*head entity*, *relation*, *tail entity*). Given a set *T* of triples (*h*, *r*, *t*) composed of two entities *h*, *t* ∈ ***E*** (the set of entities), a relation *r* ∈ ***R*** (the set of relations), descriptions for the corresponding two entities *h_d_*, *t_d_* ∈ ***ED*** (the set of entity descriptions), and a description for the corresponding relation *r_d_* ∈ ***RD*** (the set of relation descriptions), KGED learns vector embeddings of the entities, relations, and their descriptions. Herein, embeddings for entities and relations are set as $h,r,t\in R^{k}$, and embeddings for descriptions for two entities and relations are set as $h_{d},r_{d},t_{d}\in R^{l}$.

A CNN is a multilayer learning framework for learning nonlinear features to capture complex relationships. These consist of an input layer, a convolutional layer, and an output layer for logistic regression. In KGED, the input layer consists of the matrix for the triple and the corresponding descriptions. The former can be represented as the matrix $A=[h,r,t]\in R^{k\times 3}$, and the latter is denoted as the matrix $B=[h_{d},r_{d},t_{d}]\in R^{l\times 3}$. Herein, we define that ***A_i:i+k-1_*** and ***B_i:i+l-1_*** refer to the concatenation of row (***A_i_***, ***A_i+1_***,…, ***A_i+k-1_***) and (***B_i_***, ***B_i+1_***,…, ***B_i+l-1_***), respectively, where *i* starts with 1.

On the convolution layer we used the filter $\omega \in R^{n\times 3}$, which is repeatedly applied to a window of *n* rows of two input matrices to create feature maps containing highly informative features describing the inputs. Note that filter sizes *n* are set to 3, 4, and 5 in this study. For example, a feature *α_i_* is generated from a window of *n* rows of the input matrix for the triple by

$$\begin{array}{c} \alpha_{i}=g(\omega_{1}\cdot A_{i:i+n-1}+b_{1}). \end{array}$$ (3)

Similarly, a feature *β_i_* is produced from a window of *n* rows of the input matrix for the corresponding descriptions by

$$\begin{array}{c} \beta_{\text{i}}=g(\omega_{2}\cdot B_{i:i+n-1}+b_{2}) \end{array}$$ (4)

where $b_{1},b_{2}\in R$ are bias terms and *g* is a nonlinear activation function, such as the hyperbolic tangent and rectified linear unit (ReLU). Each filter (***ω***_1_, ***ω***_2_) is respectively applied to a window of *n* rows in the two input matrices, (***A_1:n_***, ***A_2:n+1_***,…, ***A_k-n+1:k_***) and (***B_1:n_***, ***B_2:n+1_***,…, ***B_l-n+1:l_***) to generate each corresponding feature map ***α*** and ***β*** as:

$$\begin{array}{c} \alpha =[\alpha_{1},\alpha_{2},\ldots ,\alpha_{k-n+1}], \end{array}$$ (5)

$$\begin{array}{c} \beta =[\beta_{1},\beta_{2},\ldots ,\beta_{l-n+1}] \end{array}$$ (6)

with $\alpha \in R^{(k-n+1)\times 1}$ and $\beta \in R^{(l-n+1)\times 1}$.

In KGED, different filters are used to generate different feature maps. Let **Θ** and *δ* represent the set of filters and the number of filters. Then, there are *δ* feature maps such as *δ* = |**Θ**|. These *δ* feature maps for each filter size (*n* = 3, 4, 5) are concatenated into a single vector. Two concatenated vectors, namely $X\in R^{3\delta (k-3)\times 1}$ for the triple and $Y\in R^{3\delta (l-3)\times 1}$ for its corresponding descriptions, are generated. Thereafter, these two vectors are also concatenated and calculated with a weight vector ***w*** in $R^{3\delta (k+l-6)\times 1}$ via a dot product to derive a score for a given triple *(h, r, t)*.

Herein, we define the score function *f* of KGED as follows:

$$\begin{array}{c} \begin{array}{cc} & f(h,r,t)=concat(X,Y)\cdot w,\\ & X=concat(g([h,r,t]*\Theta_{1})),\\ & Y=concat(g([h_{d},r_{d},t_{d}]*\Theta_{2})) \end{array} \end{array}$$ (7)

where **Θ**_1_, **Θ**_2_ and ***w*** are shared parameters. * represents a convolution operator with the set of filters, and *concat* represents the operation that concatenates vectors. ***X*** and ***Y*** are concatenated vectors from feature maps for the triple and the corresponding descriptions, respectively.

To train KGED, we regularized the model by using dropout on the feature maps after the convolution operation. We also used the Adam optimizer [38], a gradient descent optimization function with an adaptive value learning rates for each parameter [39], by minimizing the loss function L with *L*_2_ regularization on the weight vector w of the model. The loss function is defined as follows:

$$\begin{array}{c} \begin{array}{cc} & L=\sum_{\left(h,r,t\right)\in \left\{T\cup T^{\prime }\right\}}\text{log}\left(1+exp\left(l_{\left(h,r,t\right)}\cdot f\left(h,r,t\right)\right)\right)\\ & +\frac{\lambda }{2}{\left\|w\right\|}_{2}^{2} \end{array} \end{array}$$ (8)

where *l*_(*h,r,t*)_ is 1 for *(h, r, t)* ∈ *T* and −1 for *(h, r, t)* ∈ *T*′, λ is the *L*_2_-regularizer to lessen over-fitting, and

$$\begin{array}{c} T^{\prime }=\left\{\left(h^{\prime },r,t\right)|h^{\prime }\in E\right\}\;\cup \;\left\{\left(h,r,t^{\prime }\right)|t^{\prime }\in E\right\}. \end{array}$$ (9)

In Eq (9), *T*′ is a set of invalid triples generated by corrupting valid triples in *T*. The set of corrupted triples is created from the training triples by replacing the head or tail entity with all the other entities in turn (but not both at the same time). The loss function prefers lower values for the training triples rather than for the corrupted triples.

## Results

### Biological knowledge graph dataset

We compared the performance of KGED with that of TransE and ConvKB (baseline model). Thus, the biological triples in Table 2 were used to train and evaluate the models. The structure of our biological knowledge graph is very complex. The reference [40] indicates that the number of averaged triples per entity (ATPE) measures the diversity and complexity of datasets, which is calculated by the total number of triples divided by the total number of entities. In general, more triples result in more complex knowledge graph structures. The performance of knowledge graph embedding models is not satisfactory when a dataset with higher ATPE is used. FB15K is a subset of FreeBase containing 14,951 entities and 1,345 different relations in 592,213 triples. WN18 is a subset of WordNet consisting of 40,743 entities and 18 relations in 151,442 triples. The ATPE values for FB15K and WN18 are 39.61 and 3.71, respectively, and the ATPE value for our biological knowledge graph with 103,625 entities and 5 relations in 3,273,215 triples is 31.59. Thus, our biological knowledge graph is as complex as FB15K.

Moreover, we observed a difference between FreeBase/WordNet and our biological KBs. First, transitive relations (if (x,y)∈R and (y,z)∈R imply (x,z)∈R) can be established in FreeBase and WordNet. However, this is not always satisfied in the biological KBs due to the characteristics of biological entities and relations between them. For example, the relation (*gene*_1_, *relate*, *gene*_3_) is not always valid, although BioGrid contains the following two gene interactions: (*gene*_1_, *relate*, *gene*_2_) and (*gene*_2_, *relate*, *gene*_3_). Second, FreeBase and WordNet consist of 1345 and 18 distinct relations, respectively, indicating that the distinction between relations is quite clear. However, relations in our biological KBs are semantically related with each other, as four among five relations are the “relate” relations (i.e., (*chemical, relate, gene*), (*chemical, relate, disease*), (*gene, relate, gene*), (*disease, relate, gene*)). We assume that these properties of biological KBs could result in relatively lower performance quality of various knowledge graph embedding models compared with using other common knowledge bases, such as FreeBase and WordNet.

### Evaluation protocol

A KB is a collection of fact triples, meaning that there is no negative triple. KBs in various domains are incomplete as they do not cover the full spectrum of knowledge. This implies that it is hard to determine whether undiscovered knowledge not currently included in the KBs is a true negative. Thus, general evaluation methods like the area under the curve between true-positive and false-positive data are unsuitable for evaluating knowledge graph embedding models. In this study, we used the same evaluation metrics including mean rank (MR) and Hits@10, as described in [21].

In the KB completion or link prediction task, the knowledge graph embedding model aims to predict a missing entity given a relation and another entity. More specifically, it infers a head entity *h* given a relation and a tail entity (*r*, *t*) or infers a tail entity *t* given a head entity and a relation (*h*, *r*). We first assessed the performance of the knowledge graph embedding models using the following ranking task: for all test triples (*h*, *r*, *t*), (1) the head entity *h* was removed and replaced by all entities except the head entity to generate the corrupted triples; (2) scores for these corrupted triples and the test triple were calculated using a score function;, (3) score values are were sorted in descending order;, and (4) the correct head entity in the test triple was recorded to obtain its rank. An identical process was repeated for predicting the tail entity. For example, let us suppose that we have the following test triple: (*chemical*_1_, *relate*, *gene*_1_). The head entity *chemical*_1_ is replaced with all chemical entities except *chemical*_1_ to generate the corrupted triples. We then calculate score values for these corrupted triples and the test triple, thus obtaining the rank of *chemical*_1_ by sorting scores in descending order. The same procedure is conducted for determining the ranking of *gene*_1_. Note that we used the “Filtered” setting [21] during evaluation, implying that any corrupted triples that already appear in our biological KBs are excluded. On the basis of the above ranking task, the mean rank (MR) is defined as the average ranking for all test triples. Hits@10 is the proportion of correct triples ranked in the top 10. Therefore, lower MR or higher Hits@10 reflects better performance on the link prediction task.

### Implementation details

#### TransE

For experiments with TransE, we selected the embedding dimension *k* ∈ {20, 50}, the SGD learning rate *lr* ∈ {0.01, 0.001, 0.0001}, *L*_1_-norm, or *L*_2_-norm, and the margin *γ* ∈ {1, 2, 5}. We fixed the batch size to 100. Training time was set to 200 epochs. Optimal configurations on our biological KBs were as follows: *k* = 50, *lr* = 0.01, *L*_1_-norm, and *γ* = 2.

#### ConvKB and KGED

For experiments with ConvKB and KGED, we chose the embedding dimension *k* ∈ {20, 50}, the learning rate *lr* ∈ {0.01, 0.001, 0.0001}, the number of filters *δ* ∈ {100, 300, 500}, and drop-out rate ∈ {0.3, 0.5, 0.7} to avoid overfitting during the training of the considered models. We fixed the batch size to 128, filter size = {3, 4, 5}, *L*_2_-regularizer λ = 0.0001, and learning epochs = 200. Optimal configurations for both models were as follows: *k* = 50, *lr* = 0.0001, *δ* = 500, and drop-out rate = 0.7. We used ReLU as the activation function *g*. To initialize the embeddings for entities and relations, we pre-trained TransE for 3,000 epochs. For KGED, we initialized the embeddings for descriptions using the universal sentence encoder. The embedding dimension for descriptions (*l*) was fixed at 512. In ConvKB and KGED, we used a single convolutional layer architecture to reduce the number of parameters in the model.

### Performance comparison of KGED with other existing models on the basis of mean rank and hits at 10

To train and evaluate the knowledge graph embedding models including TransE, ConvKB, and KGED, we randomly selected 250 test triples and 500 validation triples for each relation from 3,273,215 triples, yielding 1,250 test and 2,500 validation triples. The rest of the data were used as training triples as shown in Table 2. In this experiment, to add statistical significance to the results, we repeated this process 10 times and reported the average results in Tables 3 and 4. Note that the test and validation triples in each process are not duplicated.

**Table 3:** Comparison of the average performance values of the different knowledge graph embedding models based on the mean rank scores.

Model	Relation	Mean Rank for head ± SD (the top %)	Mean Rank for tail ± SD (the top %)	Mean Rank for both head and tail ± SD
TransE	ALL	1156.3 ± 89.9	1853.2 ± 90.2	1504.8 ± 58.7
	chemical, relate, gene	435.3 ± 60.3 (2.85%)	3760.4 ± 471.8 (5.5%)	2097.9 ± 246.3
	chemical, relate, disease	1354.1 ± 218.4 (8.87%)	397.7 ± 68.6 (3.53%)	875.9 ± 93.5
	gene, relate, gene	1691.1 ± 187.7 (2.47%)	1637.4 ± 202.9 (2.4%)	1664.3 ± 185.3
	disease, relate, gene	1222.7 ± 201.2 (10.85%)	2775.3 ± 290.4 (4.06%)	1999 ± 148.4
	disease, have, symptom	1078.4 ± 141.1 (9.57%)	694.9 ± 98.4 (7.96%)	886.7 ± 110.6
ConvKB (base)	ALL	1063.7 ± 101.4	1799 ± 125.6	1431.4 ± 108.1
	chemical, relate, gene	396.7 ± 58.5 (2.6%)	3449.4 ± 362.1 (5.05%)	1923.1 ± 192.8
	chemical, relate, disease	1354 ± 253.6 (8.87%)	465.4 ± 72.1 (4.13%)	909.7 ± 133.6
	gene, relate, gene	1116 ± 95.6 (1.63%)	1115.5 ± 80.2 (1.63%)	1115.8 ± 64.3
	disease, relate, gene	1486 ± 293.5 (13.19%)	3285.2 ± 433.2 (4.81%)	2385.6 ± 337
	disease, has, symptom	965.6 ± 118.2 (8.57%)	679.6 ± 102.3 (7.79%)	822.6 ± 104.1
KGED	ALL	805.3 ± 88.8, ***/***	1662.1 ± 122.7, **/**	1233.7 ± 94, ***/***
	chemical, relate, gene	450.7 ± 93.7 (2.95%), −/−	3659.1 ± 542.1 (5.35%), −/−	2054.9 ± 482.9, −/−
	chemical, relate, disease	1233.9 ± 214.9 (8.08%), */*	453.2 ± 90.5 (4.02%), −/−	843.6 ± 118.4, −/−
	gene, relate, gene	444.1 ± 35.5 (0.65%), ***/***	469.6 ± 38.6 (0.69%), ***/***	456.9 ± 31, ***/***
	disease, relate, gene	1197.2 ± 209.3 (10.63%), −/*	3218.5 ± 321.2 (4.71%), −/−	2207.9 ± 212.2, −/*
	disease, has, symptom	700.9 ± 105.6 (6.22%), ***/**	510.1 ± 92.2 (5.84%), ***/***	605.5 ± 93.2, ***/***

To add statistical significance to the prediction results, we repeatedly split biological triples into the same number of training, validation, and test triples as shwon in Table 2 10 times. Here, we reported the average results. SD represents standard deviation, and the asterisks in the table indicate the number of outperforming cases compared with TransE and ConvKB (−; *; **; ***). The single asterisk (*) indicates that KGED outperformed the comparative model in 8 (out of 10) tests. The double asterisk (**) and triple asterisk (***) represent 9 and 10 (out of 10) tests, respectively. The symbol “−” indicates that KGED outperformed the comparative model in less than or equal to 7 tests.

**Table 4:** Comparison of the performance of the different knowledge graph embedding models on the basis of Hits@10 (in %).

Model	Hits@10 for head	Hits@10 for tail	Hits@10 for both head and tail
TransE	16.5	14.9	15.7
ConvKB (base)	19	17.4	18.2
KGED	27.4	26.1	26.8

Table notes the average results based on Hits@10 for each knowledge graph embedding model.

We used mean rank and hits at 10 (Hits@10) as a metric to evaluate the performance of the knowledge graph embedding models on a link prediction task. Table 3 displays the performance of each model on the link prediction task based on mean rank scores. Each knowledge graph embedding model is shown in the first column. In the second column, the name of each relation is listed to indicate the individual performance according to each relation. The name denoted as “ALL” is for indicating the average of mean ranks for the five relations. The mean rank for predicting *h* or *t* given (*r*, *t*) or (*h*, *r*) is described in the third and fourth columns, respectively. For example, in Table 3, KGED achieves a mean rank of 444.1 for the head entity (*h*) in the relation (*gene_head_, relate, gene_tail_*). This means that correct *gene_head_* entities given (*relate, gene_tail_*) pairs in the test triples were ranked in the top 0.65% on average among a total of 68,364 genes. The last column represents the mean rank for both head and tail entities ((*mean rank for head + mean rank for tail)/2*). In the KGED part in Table 3, we described the statistical significance of KGED compared with TransE and ConvKB using an asterisk symbol. As we previously mentioned, we repeatedly selected random triples for training, validation, and test data 10 times. Therefore, to calculate the statistical significance, we counted how many times KGED performed better than TransE and ConvKB for each test. According to this result, the KGED model showed remarkable performance in inferring gene-gene and disease-symptom relationships compared with the comparative models.

Table 4 represents the performance of each model based on Hits@10. Each Hits@10 for *h* and *t* is shown in the second and third columns, respectively. For example, the Hits@10 for predicting head entities in KGED was 27.4%, indicating that 27.4% of the 1250 original head entities in the test triples were correctly ranked within the top 10. The last column shows the average Hit@10 scores for both head and tail entities.

Based on the results in Tables 3 and 4, KGED achieved a significant improvement of 1504.8 − 1233.7 = 271.1 in the mean rank for both head and tail entities (approximately 18.02% improvement), and 26.8 − 15.7 = 11.1% in Hits@10 when compared with TransE. Additionally, KGED obtained 1431.4 − 1233.7 = 197.7 absolute improvement in the mean rank for both head and tail entities (approximately 13.8% improvement), and 26.8 − 18.2 = 8.6% improvement in Hits@10 when compared with ConvKB (baseline model).

Furthermore, as shown in Table 3, KGED resulted in a huge improvement of 1664.3 − 456.9 = 1207.4 in the mean rank for the relation, (*gene*, *relate*, *gene*), which is an improvement of approximately 72.6% compared with TransE. Moreover, KGED showed an improvement of approximately 59.1% in the mean rank for the same relation (1115.8 − 456.9 = 658.9) compared with ConvKB. We further focused on this improvement because gene-gene interactions are important for predicting the pathogenesis of human diseases. Therefore, gene-gene interactions inferred by KGED can be utilized for further research. In the next section, we performed describe the results of experiments performed to prove how such gene-gene interactions inferred by KGED can address an actual biological problem such as discovering genes that are closely related to a specific disease.

To prove validity of inferred relationships, we additionally investigated inferred disease-gene relationships. First of all, we collected the top 10 disease-gene relationships inferred by KGED for three cancer types (breast, lung, prostate). Then, we searched for evidence sentences in articles describing that those genes are actually related to each cancer. The results are shown in S9 Table. In breast cancer, evidence sentences in articles were found for all but two genes (TPI, ARMD14). In lung cancer, evidence sentences were also found for all but three genes (BID, NPY, TPI). In prostate cancer, we found supporting sentences for all genes. Although we could not find any supporting information about relationships between breast cancer and two genes (TPI, ARMD14) and between lung cancer and three genes (BID, NPY, TPI), it may be worthwhile to further investigate about these relationships.

### Analyzing disease-related subnetworks using different centrality measures

In [13], linear (weighted logistic regression (WLR)) and nonlinear classification (weighted kernel logistic regression (WKLR)) models were used to predict gene-gene interactions based on the co-occurrence frequency between biological terms, such as genes and Gene Ontology (GO) terms, within the biomedical literature. Using gene-gene interactions predicted by their models, they also constructed gene interaction subnetworks related to different cancer types (i,e., prostate, breast, lung). These subnetworks by were analyzed using different centrality measures to identify which genes are important or more central in the network.

In this experiment, we evaluated the ability of KGED to predict disease-gene associations using predicted gene-gene interactions and three types of cancer-related seed genes. This was done by comparing its performance with that of [13] and ConvKB. To construct disease-specific subnetworks and analyze these networks for their ability to identify disease-related human genes (with reference to two benchmarks such as MalaCards [26] and the National Cancer Institute’s Genomic Data Commons (NCI’s GDC [41]), we carried out the following steps:

1. **Inference of human gene-gene interactions**: KGED returns predicted the head or tail entities for a given pair (*r*, *t*) or (*h*, *r*), respectively. We collected 13,905 human genes in our biomedical dataset and used them to construct the input data for KGED in the form of (*head entity*, *relation*). Thus, we entered a set of pairs (*head_human_gene_i_*, *g_relate_g*) for *i* = 1, 2, …, 13,905 to KGED for predicting corresponding tail genes with inference scores. These were calculated by the score function in Eq (7). We then ranked these predictions in accordance with the inference scores. Higher ranked predictions had higher reliability than the lower ranked ones. Note that we removed (*disease, relate, gene*) triples of the three cancer types from biological KBs when inferring human gene-gene interactions for predicting cancer-gene associations.
2. **Collecting an initial list of seed genes**: The seed gene is already known to be related to a specific disease. In [13], OMIM was used to download seed genes related to prostate, breast, and lung cancer. To construct disease-related subnetworks, we used the same seed genes (18 prostate cancer seed genes, 23 for breast cancer, 16 for lung cancer) used in [13]. The seed genes for each cancer type are listed in Table 5.
3. **Constructing disease-related subnetworks**: In the previous step, we inferred human gene-gene interactions using KGED. From these predicted gene-gene relationships, we extracted all pairs that are related to at least one seed gene to construct each disease-related subnetwork. All pairs of gene-gene interactions in the generated subnetwork therefore contain at least one seed gene. Here, the subnetwork contains a subset of highly ranked gene-gene interactions when sorted by prediction scores. In this study, we performed experiments with several different numbers of gene-gene interactions from 5000 to 100,000 by every 5,000 pairs.
4. **Analyzing the subnetworks using centrality measures**: In this step, we used Cytoscape (https://cytoscape.org, version 3.7.1) [42], which is an open-source software tool for integration, visualization, and analysis of biomedical networks composed of protein, gene, and other interactions. We used this tool to rank genes in the disease-related network by calculating different centrality measures. Centrality measures determine the relative significance of a node in a network. In this study, we used the four centrality metrics closeness, betweenness, degree, and eigenvector, as follows:
  (a) *Closeness centrality* The closeness centrality is the sum of the shortest distances between a node and all the other nodes, wherein the distance from one node to another is defined as the length of the shortest path. Therefore, a node with a high closeness value is most central in the network, indicating that all other nodes can be reached easily from this node. The closeness centrality of a node *V*_i_ is defined as:
    $$\begin{array}{c} C_{C}\left(V_{i}\right)=\frac{|V|-1}{\Sigma_{V_{j}\in V}distance\left(V_{i},V_{j}\right)}, \end{array}$$ (10)
    where *V* is a set of nodes in a network, and distance(*V_i_*, *V_j_*) is the shortest distance between nodes *V_i_* and *V_j_*.
  (b) *Betweenness centrality* The betweenness centrality is defined as the number of times that a node acts as a bridge in the shortest paths between two other nodes. A high betweenness value represents its role as a gateway for connecting different parts of the network. The betweenness centrality of a node *V*_i_ is given by:
    $$\begin{array}{c} C_{B}\left(V_{i}\right)=\frac{\Sigma_{V_{i}\neq V_{j}\neq V_{k}\in V}\frac{\sigma (V_{j},V_{k}|V_{i})}{\sigma (V_{j},V_{k})}}{(|V|-1)(|V|-2)/2}, \end{array}$$ (11)
    where *σ(V_j_, V_k_)* is the number of shortest paths from node *V_j_* to node *V_k_*, and *σ(V_j_, V_k_|V_i_)* is the number of the paths that pass through node *V_i_*.
  (c) *Degree centrality* The degree centrality is based on the degree of a node. Nodes with a higher degree are more central to the network and tend to influence others more powerfully. The degree centrality of a node *V*_i_ can be calculated by:
    $$\begin{array}{c} C_{D}\left(V_{i}\right)=\frac{\left|N(V_{i})\right|}{|V|-1}, \end{array}$$ (12)
    where *N(V_i_)* is the set of nodes connected to *V_i_*.
  (d) *Eigenvector centrality* The eigenvector centrality is an extension of degree centrality. The difference is that the eigenvector centrality gives more weight to connections with more highly connected nodes. Thus, it is based on the idea that the important node has connections to other nodes that are themselves important in the network. The score of a node will therefore be higher if it is connected to nodes with a high eigenvector value than if it is connected to nodes with a low eigenvector value. The eigenvector centrality of a node *V*_i_ is defined as:
    $$\begin{array}{c} C_{E}\left(V_{i}\right)=\frac{1}{\lambda }\sum_{V_{j}\in N(V_{i})}w_{ji}\times C_{E}\left(V_{j}\right), \end{array}$$ (13)
    where *w_ji_* is the weight of the edge between nodes *V_j_* and *V_i_*, λ is a constant, and *N(V_i_)* is the set of nodes connected to *V_i_*.
  We applied the above centrality measures to the disease-related subnetworks constructed in the previous step to test the prediction quality of KGED. Furthermore, we compared KGED with the proposed approaches in [13] and ConvKB. We then enumerated the top 15 genes ranked by each centrality measure for three cancer types (prostate, breast, lung cancers) and counted how many of them appeared in the two benchmarks (MalaCards and NCI’s GDC) to calculate the precision values of each centrality measure. Note that the datasets of the two benchmarks are the same as those used in [13]. MalaCards contains 261, 317, and 239 known genes for prostate, breast, lung cancers, respectively. The NCI’s GDC includes 455 prostate genes, 562 breast genes, and 570 known genes related to lung cancer. We repeated the above experiment with ConvKB.

**Table 5:** The seed genes related to each cancer type.

Breast cancer	Lung cancer	Prostate cancer
CASP8	CASP8	PCAP
PIK3CA	PIK3CA	HPC5
SLC22A1L	SLC22A1L	MAD1L1
KRAS	KRAS	HPC4
BRCA2	FASLG	BRCA2
CDH1	DLEC1	CDH1
CHEK2	RASSF1	CHEK2
RAD54L	IRF1	HIP1
BARD1	PRKN	MSR1
HMMR	EGFR	KLF6
NQO2	BRAF	PTEN
ESR1	MAP3K8	MXI1
RB1CC1	ERCC6	CD82
TSG101	PPP2R1B	ZFHX3
ATM	ERBB2	HPCQTL19
XRCC3	CYP2A6	HPC3
AKT1		HPC6
RAD51A		AR
PALB2		
TP53		
PHB		
PPM1D		
BRIP1		

The seed genes are already known to be related to a specific disease. We used 23 breast cancer genes, 16 lung cancer genes, and 18 prostate cancer genes as seed genes.

S1 and S2 Tables show the precision of each centrality measure for the top 15 ranked genes in each cancer subnetwork, as generated by ConvKB and KGED. In both tables, the precision results are classified according to the cancer types and benchmark datasets (MalaCards, NCI’s GDC, and combining MalaCards and NCI’s GDC). Note that the first column represents the number of top *N* gene-gene interactions predicted by a given model in the subnetwork. For example, in S2 Table, KGED achieved 100% precision for all centrality measures evaluated against breast cancer genes with reference to MalaCards. Therefore, all top 15 ranked genes sorted by each centrality measure in the breast cancer subnetwork, consisting of the top 80,000 predicted gene pairs, were found to be associated to breast cancer with reference to MalaCards.

As shown in the tables, regarding the top 15 prostate-cancer-genes predicted by ConvKB and KGED, we achieved balanced and optimal results for all centrality measures evaluated against all benchmark datasets when *N* was 75,000 for ConvKB and 85,000, 90,000, 95,000, and 100,000 for KGED. Optimal precision results were obtained for the top 15 breast cancer genes when *N* was 85,000, 90,000 for ConvKB and 80,000, 85,000, 90,000, 95,000, and 100,000 for KGED. Lastly, the best precision results were obtained for the top 15 lung cancer genes when *N* was 85,000 for ConvKB and 95,000 and 100,000 for KGED. We also represent the average precision values for each centrality measure against each benchmark to compare the overall performance of the two models. When comparing the cancer types, the average precision results for prostate and breast cancer show that KGED predicted genes for prostate and breast cancer more effectively than ConvKB according to all benchmark datasets. However, ConvKB outperformed KGED in predicting lung cancer genes according to MalaCards and MalaCards+NCI’s GDC. According to the average precision values for all centrality measures and all cancer types, degree centrality is the best measure to detect most influenced nodes, and betweenness centrality is the second best. We also reported the precision results for the four centrality measures evaluated against corresponding benchmark data in S3–S5 Tables for the top *n* ranked genes predicted by KGED (N = 100,000). For each centrality measure, as *n* increased, the precision degreased, gradually converging towards each other.

Furthermore, in Tables 6–8, we summarized the performance comparison of WLR and WKLR in [13], ConvKB, and KGED. In this comparison, the balanced and optimal results for ConvKB and KGED were chosen as compare objects. In Tables 6 and 8, both ConvKB and KGED not only achieved the highest precision, but also performed better than WLR and WKLR for most of centrality measures for all cancer types when evaluated against MalaCards and MalaCards+NCI’s GDC data. Especially, the precision values achieved by all centrality measures evaluated against MalaCards and MalaCards+NCI’s GDC data were 100% for the top 15 breast cancer genes predicted by ConvKB and KGED. All 15 predicted genes are therefore related to breast cancer, with reference to the above two benchmark datasets. On the other hand, both WLR and WKLR performed slightly higher than ConvKB and KGED in many of the centrality measures for each cancer type when evaluated against the NCI’s GDC dataset in Table 7. For example, WLR correctly predicted 80% of prostate-related genes with reference to NCI’s GDC using eigenvector centrality, while the precision values of ConvKB and KGED were 66.7% and 53.3%, respectively.

**Table 6:** A comparison for the precision values of the top 15 ranked genes related to each cancer type by each centrality measure and against MalaCards and by each approach.

	Closeness	Betweenness	Degree	Eigenvector
Prostate Cancer				
WLR [13]	53.3	86.7	80	66.7
WKLR [13]	46.7	80	86.7	66.7
ConvKB [22]	93.3	100	100	93.3
KGED	100	100	100	86.7
Breast Cancer				
WLR [13]	80	86.7	93.3	93.3
WKLR [13]	46.7	100	100	86.7
ConvKB [22]	100	100	100	100
KGED	100	100	100	100
Lung Cancer				
WLR [13]	73.3	80	86.7	93.3
WKLR [13]	60	86.7	86.7	86.7
ConvKB [22]	93.3	93.3	93.3	93.3
KGED	93.3	93.3	93.3	93.3

**Table 7:** A comparison for the precision values of the top 15 ranked genes related to each cancer type by each centrality measure and against NCI’s GDC and by each approach.

	Closeness	Betweenness	Degree	Eigenvector
Prostate Cancer				
WLR [13]	80	60	66.7	80
WKLR [13]	33.3	60	60	60
ConvKB [22]	66.7	66.7	66.7	66.7
KGED	66.7	66.7	66.7	53.3
Breast Cancer				
WLR [13]	73.3	40	53.3	86.7
WKLR [13]	46.7	66.7	66.7	80
ConvKB [22]	53.3	60	53.3	60
KGED	53.3	60	60	60
Lung Cancer				
WLR [13]	20	20	33.3	86.7
WKLR [13]	40	40	40	60
ConvKB [22]	46.7	40	40	40
KGED	46.7	40	40	40

**Table 8:** A comparison of the precision values of the top 15 ranked genes related to each cancer type by each centrality measure and against both MalaCards and NCI’s GDC and by each approach.

	Closeness	Betweenness	Degree	Eigenvector
Prostate Cancer				
WLR [13]	93.3	93.3	93.3	86.7
WKLR [13]	60	86.7	93.3	80
ConvKB [22]	100	100	100	100
KGED	100	100	100	86.7
Breast Cancer				
WLR [13]	80	86.7	93.3	93.3
WKLR [13]	53.3	100	100	86.7
ConvKB [22]	100	100	100	100
KGED	100	100	100	100
Lung Cancer				
WLR [13]	73.3	80	86.7	100
WKLR [13]	66.7	86.7	86.7	93.3
ConvKB [22]	93.3	93.3	93.3	93.3
KGED	93.3	93.3	93.3	93.3

### Prostate cancer case study and comparison with recent approaches

In this section, we used another ground truth data for prostate cancer (the Prostate Gene DataBase (PGDB)) as the benchmark for evaluating the results inferred by KGED. PGDB is a curated repository of genes related to human prostate cancer. It consists of 165 unique genes, of which 129 are validated by evidence from Medline abstracts, and 36 are supported by expression data. We evaluated the quality of our system by comparing it with recent approaches [13, 14, 24, 25].

- CGDA [14]: CGDA infers disease-gene associations from biomedical literature using dependency parsing and support vector machines. It constructs a prostate cancer-specific gene-interaction network from genes known to be related to prostate cancer from predicted disease-gene associations. It then uses centrality measures to identify central genes in the network.
- EDC-EDC [24]: EDC-EDC predicts disease-gene associations from biological texts by applying novel linguistic computational techniques. This is called a hybrid constituency-dependency parser, which overcomes the limitations of current constituency and dependency parsers. It constructs a disease-specific gene interaction network and infers the gene-disease associations by using centrality measures.
- MCforGN [25]: MCforGN identifies functionally related genes on the basis of their co-occurrences in PubMed abstracts. Using these related genes, it builds a disease-specific genetic network and detects disease-gene associations by employing centrality measures.
- Rare-event classifier [13]: Rare-event classifiers predict gene-gene interactions based on their co-occurrence frequency in PubMed abstracts using their linear and nonlinear rare-event classification models. It constructs a genetic co-occurrence network for the entire human genome to extract disease-related subnetworks, and uses centrality measures to identify new candidate genes that could be connected directly to the disease.

To conduct the prostate case study, we first followed the same procedure described in the previous subsection. This included the inference of human gene-gene interactions by applying KGED, in addition to constructing a prostate cancer-related gene-interaction subnetwork using 18 prostate cancer seed genes downloaded from OMIM. We then evaluated the performance of KGED when detecting prostate cancer-related genes. This was done by comparing its performance with that of recent approaches, as aforementioned. We used the ground truth data PGDB as the benchmark. We applied closeness, betweenness, and degree centrality measures to rank the genes in the prostate cancer-related gene-interaction subnetwork. We then checked how many of top 10 ranked genes by each centrality measure appeared in the PGDB benchmark dataset, allowing us to calculate the precision of KGED. Note that the same procedure described above was conducted for ConvKB.

Table 9 shows the precision of the top 10 ranked genes associated with prostate cancer based on PGDB by each centrality measure by ConvKB and KGED and by *N*, the number of gene-gene interactions that comprise the subnetwork. For the top 10 ranked genes by ConvKB, the highest precision values by closeness, betweenness, and degree were 70%, 70%, and 90%, respectively, and the balanced performance of ConvKB was obtained when *N* = 80,000, 85,000, 95,000, 100,000. Moreover, KGED achieved the highest precision values of 90% for closeness, 80% for betweenness, and 90% for degree centrality. For example, Table 10 shows the top 10 genes by degree centrality measure by KGED and ConvKB when *N* = 40,000. In the table, the degree represents the degree of the corresponding gene in the subnetwork. In KGED, all genes but HIP1 were PGDB genes. However, in ConvKB, three genes were not PGDB genes. It means that the gene-gene interactions in the subnetwork created using KGED have more connections with PGDB genes. Taken as a whole, KGED achieved average precision scores of 67.5% for closeness, 74.5% for betweenness, and 82.5% for degree centrality. These values are 5%, 10.5%, and 8% higher than ConvKB, respectively. The results in Table 9 therefore show that KGED performed better than ConvKB, and the best performance of KGED for all centrality measures was obtained when the number of gene-gene interactions comprising the subnetwork (*N*) was set to 40,000.

**Table 9:** The precision values of the top 10 ranked genes associated with prostate cancer based on PGDB by each centrality measure, by ConvKB and KGED and by the number of inferred gene-gene interactions that makes up the subnetwork.

*N*	Closeness		Betweenness		Degree	
	ConvKB	KGED	ConvKB	KGED	ConvKB	KGED
5000	30	40	70	80	80	80
10000	50	20	70	80	90	80
15000	50	30	70	80	90	80
20000	50	40	70	80	90	90
25000	60	50	60	80	90	90
30000	50	60	60	80	70	90
35000	50	60	60	80	70	90
40000	70	90	60	80	70	90
45000	70	80	60	80	70	80
50000	70	80	60	70	70	80
55000	70	80	60	70	70	80
60000	70	80	60	70	70	80
65000	70	80	60	70	70	80
70000	70	80	60	70	70	80
75000	70	80	60	70	70	80
80000	70	80	70	70	70	80
85000	70	80	70	70	70	80
90000	70	80	60	70	70	80
95000	70	80	70	70	70	80
100000	70	80	70	70	70	80
[*Avg*.]	62.5	67.5	64	74.5	74.5	82.5

**Table 10:** The top 10 ranked genes by degree centrality measure by KGED and ConvKB when *N* = 40,000. The columns of PGDB represent whether these genes are PGDB genes or not.

KGED			ConvKB		
Degree	Gene Name	PGDB	Degree	Gene Name	PGDB
7168	CDH1	YES	10458	CDH1	YES
5372	MAD1L1	YES	8045	MAD1L1	YES
4235	HIP1	NO	7997	MSR1	NO
4175	AR	YES	7569	AR	YES
3828	MXI1	YES	7148	MXI1	YES
3501	PTEN	YES	7071	HIP1	NO
3347	BRCA2	YES	6439	ZFHX3	NO
2449	KLF6	YES	6205	CD82	YES
2295	CD82	YES	6106	BRCA2	YES
2202	PCAP	YES	5747	PTEN	YES

In S3–S5 Figs, we show the precision-recall (PR) curve of the top *n* ranked genes according to each centrality measure obtained using ConvKB (*N* = 80,000) and KGED (*N* = 40,000). In order to draw the PR curve, we calculated a set of precision and recall values for every top *n*, where *n* starts with 10 and increases by 10. Note that the recall measure was computed by dividing the number of PGDB genes occurring in the top *n* genes ranked by each centrality measure over the total number of PGDB genes. As shown in the figures, precision values were the highest for the top 10 and then decreased as *n* increased. In contrast, recalls were the lowest for the top 10 and then increased as *n* increased. For example, precision and recall for the top 10 genes by closeness were 90% and 11.4% (black bold line) for KGED and 70% and 8.86% (black dash line) for ConvKB, respectively. For each centrality measure, both precision and recall values of KGED for genes in high ranks were relatively higher than those of ConvKB and converged towards each other as the rank *n* increased. Since the top ranked genes were considered more important, we concluded that KGED was more suitable than ConvKB for identifying disease-related genes based on the above results.

In Table 11, we also compared the performance of KGED with the existing models for inferring genes related to prostate cancer on the basis of PGDB. As described in the table, the performance of KGED is comparable with other models. KGED has correctly predicted 90% of prostate cancer genes related to PGDB by closeness centrality, which is a performance improvement of 10% to 20% over all four comparison models. The precision of KGED by betweenness centrality was 80%, which is relatively lower than CGDA, EDC-EDC, and MCforGN. Conversely, KGED achieved the best precision score (90%) by degree centrality compared with all other models. Note that only two models, including KGED and CGDA, achieved the highest precision (90%). However, KGED detected the global importance of a node in the network more effectively than CGDA, as closeness centrality is a global topological characteristic of the network [43]. Overall, our system achieved well-balanced precision scores via all centrality measures. In particular, closeness and degree centrality measures showed the best performance.

**Table 11:** A comparison for the precision of the top 10 ranked genes associated with prostate cancer based on PGDB, by each centrality measure and by each existing model.

	Closeness	Betweenness	Degree
CGDA [14]	70	90	80
EDC-EDC [24]	77.3	86.4	82.8
MCforGN [25]	78	83	82
Rare-event classifier [13]	80	80	80
KGED	90	80	90

Additionally, we conducted an experiment to see how the number of prostate cancer-related seed genes used for constructing the subnetworks affected the precision scores. Since the seed genes were already known to be related to a specific disease and connected to various other genes, we assumed that the precision scores would be influenced by the number of seed genes. Thus, we measured the precision values of the top 10 genes using different numbers of prostate seed genes, *sn* = 8, 10, 12, where *sn* indicates the number of seed genes. For each *sn*, we randomly selected prostate seed genes five times since the importance of different genes in cancers may vary. Using different numbers of seed genes, we analyzed the subnetworks to calculate the precision scores. In S6–S8 Tables, according to the number of seed genes, we describe the average precision values of the top 10 ranked genes based on PGDB genes by each centrality measure. Compared with the result using all prostate seed genes shown in Table 9, smaller the number of seed genes (*sn*), lower the average precision scores. Seed genes are central genes in the subnetwork, meaning that they significantly affect the disease-related subnetwork. Therefore, based on these results, we concluded that the number of seed genes affected the precision scores.

## Discussion

As shown in Tables 3 and 4, ConvKB and KGED outperformed TransE. The two former models were based on convolutional neural networks, and the latter was the direct translation model. Here, we investigated why a standard CNN learning method had benefits for embedding the biological knowledge graph compared with the traditional knowledge graph embedding model. Because CNN is specialized to extract useful localized features from a low-dimensional vector space, we assumed that the representations of entities in the knowledge graph trained by ConvKB were different from those trained by TransE. The cosine distance represented the semantic similarity between two embedding vectors. To identify the differences between the representations of entities trained by TransE and those trained by ConvKB, we calculated cosine distances between embedding vectors for each head and tail entity in each training triple, which were trained by TransE and ConvKB, depending on the window of *n* rows of embedding.

S1 Fig shows the distribution of cosine distances between the trained embedding vectors of head and tail entities (ie, $h\in R^{50\times 1}$ and $r\in R^{50\times 1}$) contained in each set of fact and negative (or corrupted) triples in the training data when the window size *n* was 50. In the figure, the red and gray bars represent the results of TransE and ConvKB, respectively. Additionally, the bold and dashed bars indicate the results obtained when we used fact and negative triples, respectively. In the graph, the x-axis is a cosine distance range between 0 and 1 with 1 representing the maximum possible distance, ie, no interdependence, and the y-axis is the ratio of the number of pairs of the head and tail embedding vectors (*n* dimension) whose cosine distance belongs to the corresponding range. As described in the figure, 43.78% of pairs of the head and tail embedding vectors trained by TransE were located in the cosine distance range between 0 and 0.1, which is 3.7% higher than the result from ConvKB. Similarly, the dashed bar shows the distribution of cosine distances using negative triples generated during training. In the negative triples, the head and tail entities are supposed to be semantically unrelated. In the figure, compared with the results obtained by ConvKB (dashed red bars), the number of pairs of the head and tail embedding vectors trained by TransE (dashed gray bars) were distributed more on the right side of the graph, meaning that both head and tail entities in negative triples were more properly trained because they were semantically unrelated. For the whole embedding vectors, TransE produced better embedding representations for entities in the knowledge graph than ConvKB.

However, unlike results from the whole embedding vectors, when the same experiments were performed using trained local embeddings of head and tail entities, results were different, when *n* was 3, 4, or 5. For example, we collected each set of 48 local embeddings by sliding the window (e.g., *n* = 3) across $h\in R^{50\times 1}$ and $r\in R^{50\times 1}$. We then calculated cosine distances between each pair of local embeddings from ***h*** and ***t***. S2 Fig shows the average distribution of cosine distances between trained embeddings vectors of head entities and those of tail entities contained in each set of fact and negative triples in the training data when *n* was 3, 4, or 5. Note that we averaged each distribution for *n* = 3, 4, or 5 to generate S2 Fig. According to the figure, more pairs of local embeddings were distributed in the cosine distance range between 0 and 0.1 when we used ConvKB compared with TransE. Thus, from this perspective, ConvKB produced better local embedding vectors for entities in the knowledge graph. Furthermore, dashed bars in gray and red showed the distributions resulted by TransE and ConvKB, respectively, when using trained local embeddings of head and tail entities in the negative triples. Although the head and tail entities in the negative triple were semantically unrelated, the ratio of the number of pairs of local embeddings in the cosine distance range between 0 and 0.1 was significantly higher when we used ConvKB than when we used TransE. According to this experimental result, we assumed that this phenomenon may have improved the performance of ConvKB and KGED compared with TransE.

In this study, we split the biological dataset into 3,269,465 training triples, 2,500 validation triples, and 1,250 test triples to derive the results of Tables 3 and 4. The reason why the numbers of triples in the validation and test datasets were relatively smaller than those in the training dataset is that it took a long time to obtain predictions for a single test triple (46.05 seconds with a GTX TITAN X graphic card). Thus, to add statistical significance to the results, we randomly selected 250 test and 500 validation triples for each relation type 10 times and summarized the statistical analysis results for the performance comparison in Tables 3 and 4.

Lastly, there are studies suggesting that Markov chains or classical statistical approaches can show more reliable results than neural networks in the field of biology and medicine [44]. In this study, our method was developed based on neural networks to incorporate biological knowledge graph, but it would be interesting to apply other approaches such as the spectral forecast model [44] instead of neural networks.

## Conclusion

In this study, we proposed a CNN-based KGED model to infer biological relationships, because the current knowledge graph-embedding models are optimized for general-purpose KBs such as FreeBase and WordNet. KGED jointly embeds both the biological triples and those of textual descriptions for biological entities. To train and evaluate the performance of KGED, we first collected a large set of biological KBs and their descriptions from well-known biological public databases. We constructed a total of 3,273,215 biological KBs (or triples) observed among biological entities such as chemicals, genes, diseases, and symptoms. We also compared the performance of KGED with that of other existing knowledge graph-embedding models such as TransE and ConvKB in inferring biological relationships. In this process, we used the mean rank and Hits@10 measurements as performance metrics. In this comparison, KGED outperformed both TransE and ConvKB with respect to the average of mean ranks and Hits@10 score. In particular, our KGED model achieved significant improvement in inferring gene-gene interactions, with 72.6% and 59.1% performance improvement in the mean rank compared to that of TransE and ConvKB, respectively. We emphasize that this improvement is meaningful because identifying undiscovered gene-gene interactions is essential for understanding the pathogenesis of various diseases.

We performed additional experiments to further validate how gene-gene interactions inferred by KGED can address actual biological problems such as finding genes closely related to human diseases. In the first experiment, we analyzed disease-related subnetworks using different centrality measures. We constructed each cancer-related subnetwork using gene-gene interactions inferred by ConvKB and KGED, respectively. We then analyzed each network using different centrality measures to identify the genes that emerge as most important or more central in the network. We compared the performance of our KGED model with that of ConvKB and two previous studies (WLR and WKLR) by comparing disease-gene related benchmarks such as the MalaCards database and the NCI’s GDC with the top 15 ranked genes based on centrality measures. Compared with the MalaCards and MalaCards+NCI’s GDC benchmark data, both KGED and ConvKB achieved the highest precision values using most centrality measures for all cancer types. In the second experiment, we focused on prostate cancer as a case study to evaluate the quality of KGED by comparing its performance with those of recently developed approaches such as CGDA, EDC-EDC, MCforGN, and rare-event classifier. We used another ground-truth dataset for prostate cancer (the Prostate Gene DataBase (PGDB)) as the benchmark for evaluating the results inferred by KGED. As a result, KGED showed well-balanced and comparable results, especially with respect to the precision values obtained by closeness and degree centrality measures. These findings indicate that our proposed method is useful and has the potential to predict candidate genes related to human diseases. Therefore, based on the experimental results, we conclude that new gene-gene interactions inferred by KGED can be helpful for future research, such as that aimed at understanding undiscovered pathogenic mechanisms of human diseases, and contribute to the field of disease treatment discovery.

## Supporting information

S1 Fig The distribution of cosine distances between the trained embedding vectors of head entities and those of tail entities contained in each set of fact and negative triples when *n* = 50. (TIFF)

S2 Fig The average distribution of cosine distances between trained embedding vectors of head entities and those of tail entities contained in each set of fact and negative triples when *n* = 3,4,5. (TIFF)

S3 Fig The precision and recall of top n ranked genes associated with prostate cancer based on PGDB by closeness centrality measure, by ConvKB (*N* = 80,000) and KGED (*N* = 40,000). Note that *n* starts at 10 and increases by 10.
(TIFF)

S4 Fig The precision and recall of top n ranked genes associated with prostate cancer based on PGDB by betweenness centrality measure, by ConvKB (*N* = 80,000) and KGED (*N* = 40,000). Note that *n* starts at 10 and increases by 10.
(TIFF)

S5 Fig The precision and recall of top *n* ranked genes associated with prostate cancer based on PGDB by degree centrality measure, by ConvKB (*N* = 80,000) and KGED (*N* = 40,000). Note that *n* starts at 10 and increases by 10.
(TIFF)

S1 Table The precisions of the top 15 ranked genes related to each cancer type by four centrality measures using ConvKB. (XLSX)

S2 Table The precisions of the top 15 ranked genes related to each cancer type by four centrality measures using KGED. (XLSX)

S3 Table The precision of top *n* ranked genes related to each cancer type, predicted by KGED (*N* = 100,000), by each centrality measure and against MalaCards. (XLSX)

S4 Table The precision of top *n* ranked genes related to each cancer type, predicted by KGED (*N* = 100,000), by each centrality measure and against NCI’s GDC. (XLSX)

S5 Table The precision of top *n* ranked genes related to each cancer type, predicted by KGED (*N* = 100,000), by each centrality measure and against both MalaCards and NIC’s GDC. (XLSX)

S6 Table The average precisions of the top 10 ranked genes associated with prostate cancer based on PGDB by closeness centrality measure. A different number of seed genes (i.e., *sn* = 8, 10, 12) was used.
(XLSX)

S7 Table The average precisions of the top 10 ranked genes associated with prostate cancer based on PGDB by betweenness centrality measure. A different number of seed genes (i.e., *sn* = 8, 10, 12) was used.
(XLSX)

S8 Table The average precisions of the top 10 ranked genes associated with prostate cancer based on PGDB by degree centrality measure. A different number of seed genes (i.e., *sn* = 8, 10, 12) was used.
(XLSX)

S9 Table The examination of evidence sentences supporting top 10 genes related to three cancer types, inferred by KGED. (XLSX)

## References

[1] Bray F, Ferlay J, Soerjomataram I, Siegel RL, Torre LA, Jemal A. Global cancer statistics 2018: GLOBOCAN estimates of incidence and mortality worldwide for 36 cancers in 185 countries. CA: a cancer journal for clinicians. 2018 68(6):394–424. 30207593 10.3322/caac.21492

[2] Stankov Karmen. Bioinformatic tools for cancer geneticists. Archive of Oncology. 2005 13(2):69–75. doi: 10.2298/AOO0502069S

[3] Campbell PJ, Getz G, Stuart JM, Korbel JO, Stein LD. Pan-cancer analysis of whole genomes. Nature. 2020 578:82–93. doi: 10.1038/s41586-020-1969-6 32025007 PMC7025898

[4] Schena M, Shalon D, Davis RW, Brown PO. Quantitative monitoring of gene expression patterns with a complementary DNA microarray. Science. 1995 270(5235):467–470. doi: 10.1126/science.270.5235.467 7569999

[5] Vaishali PK, Dr. Vinayababu A. Application of microarray technology and softcomputing in cancer biology: a review. International Journal of Biometrics and Bioinformatics (IJBB). 2011 5(4):225–233.

[6] Jin D, Lee H. FGMD: A novel approach for functional gene module detection in cancer. PloS ONE. 2017 12(12):e0188900. doi: 10.1371/journal.pone.0188900 29244808 PMC5731741

[7] Lawrence MS, et al. Mutational heterogeneity in cancer and the search for new cancer-associated genes. Nature. 2013 499(7457):214–218. doi: 10.1038/nature12213 23770567 PMC3919509

[8] Kumar RD, Swamidass SJ, Bose R.R. Unsupervised detection of cancer driver mutations with parsimony-guided learning. Nature genetics. 2016 48(10):1288–1294. doi: 10.1038/ng.3658 27618449 PMC5328615

[9] Han Y, Yang J, Qian X, Cheng WC, Liu SH, Hua X, et al. DriverML: a machine learning algorithm for identifying driver genes in cancer sequencing studies. Nucleic acids research. 2019 47(8):e45. doi: 10.1093/nar/gkz096 30773592 PMC6486576

[10] Cho A, Shim JE, Kim E, Supek F, Lehner B, Lee I. MUFFINN: cancer gene discovery via network analysis of somatic mutation data. Genome biology. 2016 17(1):129. doi: 10.1186/s13059-016-0989-x 27333808 PMC4918128

[11] Mattingly CJ, Rosenstein MC, Colby GT, Forrest JN, Boyer JL. The Comparative Toxicogenomics Database (CTD): a resource for comparative toxicological studies. Journal of Experimental Zoology Part A: Comparative Experimental Biology. 2006 305(9):689–692. doi: 10.1002/jez.a.307 16902965 PMC1586110

[12] Stark C, Breitkreutz BJ, Reguly T, Boucher L, Breitkreutz A, Tyers M. BioGRID: a general repository for interaction datasets. Nucleic acids research. 2006 34(suppl_1):D535–D539. doi: 10.1093/nar/gkj109 16381927 PMC1347471

[13] Al-Aamri A, Taha K, Homouz D, Al-Hammadi Y, Maalouf M. Analyzing a co-occurrence gene-interaction network to identify disease-gene association. BMC bioinformatics. 2019 20(1):70. doi: 10.1186/s12859-019-2634-7 30736752 PMC6368766

[14] Ozgur A, Vu T, Erkan G, Radev DR. Identifying gene-disease associations using centrality on a literature mined gene-interaction network. Bioinformatics. 2008 24(13):i277–i285. doi: 10.1093/bioinformatics/btn182 18586725 PMC2718658

[15] Szumlanski S, Gomez F. Automatically acquiring a semantic network of related concepts. Proceedings of the 19th ACM international conference on Information and knowledge management. 2010 19–28.

[16] Bollacker K, Evans C, Paritosh P, Sturge T, Taylor J. Freebase: a collaboratively created graph database for structuring human knowledge. Proceedings of the 2008 ACM SIGMOD international conference on Management of data. 2008 1247–1250.

[17] Miller G. WordNet: a lexical database for English. Communications of the ACM. 1995 38(11):39–41. doi: 10.1145/219717.219748

[18] Suchanek FM, Kasneci G, Weikum G. Yago: A core of semantic knowledge unifying wordnet and wikipedia. 16th International World Wide Web Conference, WWW. 2007 697–706.

[19] Auer S, Bizer C, Kobilarov G, Lehmann J, Cyganiak R, Ives Z. Dbpedia: A nucleus for a web of open data. The semantic web. 2007 722–735. doi: 10.1007/978-3-540-76298-0_52

[20] Vrandecic D, Krotsch M. Wikidata: a free collaborative knowledgebase. Communications of the ACM. 2014 57(10):78–85. doi: 10.1145/2629489

[21] Bordes A, Usunier N, Garcia-Duran A, Weston J, Yakhnenko O. Translating embeddings for modeling multi-relational data. Advances in neural information processing systems. 2013 2787–2795.

[22] Nguyen DQ, Nguyen TD, Nguyen DQ, and Phung D. A novel embedding model for knowledge base completion based on convolutional neural network. Proceedings of the 2018 Conference of the North American Chapter of the Association for Computational Linguistics: Human Language Technologies (NAACL). 2018 2:327–333.

[23] Choi W, Lee H. Inference of Biomedical Relations Among Chemicals, Genes, Diseases, and Symptoms Using Knowledge Representation Learning. IEEE Access. 2019 7:179373–179384. doi: 10.1109/ACCESS.2019.2957812

[24] Taha K. Extracting various classes of data from biological text using the concept of existence dependency. IEEE journal of biomedical and health informatics. 2015 19(6):1918–1928. doi: 10.1109/JBHI.2015.2392786 25616086

[25] Al-Dalky R, Taha K, Al-Homouz DD., Qasaimeh M. Applying Monte Carlo simulation to biomedical literature to approximate genetic network. IEEE/ACM transactions on computational biology and bioinformatics. 2015 13(3):494–504. doi: 10.1109/TCBB.2015.2481399 26415184

[26] Rappaport N, Nativ N, Stelzer G, Twik M, Guan-Golan Y, Iny Stein T, et al. MalaCards: an integrated compendium for diseases and their annotation. Database. 2013 bat018. doi: 10.1093/database/bat018 23584832 PMC3625956

[27] Xiang Z, Mungall C, Ruttenberg A, He Y. Ontobee: A linked data server and browser for ontology terms. Proceedings of international conference on biomedical ontology (ICBO). 2011 279–281.

[28] Rebhan M, Chalifa-Caspi V, Prilusky J, Lancet D. GeneCards: a novel functional genomics compendium with automated data mining and query reformulation support. Bioinformatics. 1998 14(8):656–664. doi: 10.1093/bioinformatics/14.8.656 9789091

[29] Wheeler DL, Barrett T, Benson DA, Bryant SH, Canese K, Chetvernin V, et al. Database resources of the national center for biotechnology information. Nucleic acids research. 2007 36(suppl_1):D13–D21. doi: 10.1093/nar/gkm1000 18045790 PMC2238880

[30] Nelson SJ, Schopen M, Savage AG, Schulman JL, Arluk N. The MeSH translation maintenance system: structure, interface design, and implementation. Medinfo. 2004 107:67–69. 15360776

[31] Hamosh A, Scott AF, Amberger JS, Bocchini CA, McKusick VA. Online Mendelian Inheritance in Man (OMIM), a knowledgebase of human genes and genetic disorders. Nucleic acids research. 2005 33(suppl_1):D514–D517. doi: 10.1093/nar/gki033 15608251 PMC539987

[32] Ayme S, Rath A, Bellet B. WHO International Classification of Diseases (ICD) Revision Process: incorporating rare diseases into the classification scheme: state of art. Orphanet journal of rare diseases. 2010 5(1):P1. doi: 10.1186/1750-1172-5-S1-P1

[33] Collobert R, Weston J, Bottou L, Karlen M, Kavukcuoglu K, Kuksa P. Natural language processing (almost) from scratch. Journal of machine learning research. 2011 12:2493–2537.

[34] Cer D, Yang Y, Kong SY, Hua N, Limtiaco N, John RS, et al. Universal sentence encoder. arXiv preprint arXiv:1803.11175. 2018 Available at: http://arxiv.org/abs/1803.11175.

[35] Mikolov T, Sutskever I, Chen K, Corrado G, Dean J. Distributed representations of words and phrases and their compositionality. Advances in neural information processing systems. 2013 3111–3119.

[36] Pennington J, Socher R, and Manning CD. Glove: Global vectors for word representation. Proceedings of the 2014 conference on empirical methods in natural language processing (EMNLP). 2014 1532–1543.

[37] Bojanowski P, Grave E, Joulin A, Mikolov T. Enriching word vectors with subword information. Transactions of the Association for Computational Linguistics. 2017 5:135–146. doi: 10.1162/tacl_a_00051

[38] Kingma D, Ba J. Adam: A method for stochastic optimization. arXiv preprint arXiv:1412.6980. 2014 Available at: https://arxiv.org/abs/1412.6980.

[39] Sutskever I, Martens J, Dahl G, G. Hinton G. On the importance of initialization and momentum in deep learning. International Conference on Machine Learning (ICML). 2013 28:1139–1147.

[40] Xiao H, Huang M, Hao Y, Zhu X. TransA: An adaptive approach for knowledge graph embedding. arXiv preprint arXiv:1509.05490. 2015 Available at: https://arxiv.org/abs/1509.05490.

[41] Xiao H, Huang M, Hao Y, Zhu X. The NCI’s Genomic Data Commons (GDC). Available at: https://gdc.cancer.gov.

[42] Shannon P, Markiel A, Ozier O, Baliga NS, Wang JT, Ramage D, et al. Cytoscape: a software environment for integrated models of biomolecular interaction networks. Genome research. 2003 13(11):2498–2504. doi: 10.1101/gr.1239303 14597658 PMC403769

[43] Liu J, Xiong Q, Shi W, Shi X, Wang K. Evaluating the importance of nodes in complex networks. Physica A: Statistical Mechanics and its Applications. 2016 452:209–219. doi: 10.1016/j.physa.2016.02.049

[44] Gagniuc PA, Ionescu-Tirgoviste C, Gagniuc E, Militaru M, Nwabudike LC, Pavaloiu BI, et al. Spectral forecast: A general purpose prediction model as an alternative to classical neural networks. Chaos: An Interdisciplinary Journal of Nonlinear Science. 2020 30(3):033119. doi: 10.1063/1.5120818 32237773

